# Bio-Exercise (BioEx) - A biocreative orofacial myofunctional therapy: preliminary cephalometric study and clinical application

**DOI:** 10.1590/2177-6709.27.2.e2220367.oar

**Published:** 2022-05-23

**Authors:** Li-In LIM, HyeRan CHOO, Luiz Fernando ETO, Kyu-Rhim CHUNG, Seong-Hun KIM

**Affiliations:** 1 Kyung Hee University, Department of Orthodontics (Seul, South Korea).; 2 Stanford University School of Medicine, Lucile Packard Children’s Hospital, Department of Plastic Surgery (Palo Alto/CA, USA).; 3 Exceo CMMG, Specialization in Orthodontics (Belo Horizonte/MG, Brazil).

**Keywords:** Myofunctional therapy, Orthodontic appliances, Orthodontics, interceptive, Biocreative orthodontics, Bio-exercise

## Abstract

**Objective:**

To introduce newly structured and developed orofacial myofunctional therapy (OFMFT) protocols named Bio-Exercise (BioEx), and evaluate the treatment effect of this method, using lateral cephalometric analysis on malocclusion with low tongue posture in young patients.

**Methods:**

A retrospective preliminary study was performed using orthodontic records from 28 patients (mean age of 8.41±1.45-year-old, 13 males, 15 females) treated with BioEx therapy using tongue elevators for 18.14±9.04 months (range: 6 to 37 months). Pretreatment (T_0_) and post-BioEx therapy (T_1_) lateral cephalograms were subsequently analyzed for tongue posture changes by linear, anteroposterior and vertical measurements. The data were analyzed by paired *t*-test, considering a 5% significance level.

**Results:**

The tongue length (TGL) and tongue height (TGH) increased statistically significant between T_0_ and T_1_. The decrease of the dorsum of the tongue perpendicular to the palatal plane (Td-PP value) was statistically significant. The increase of the tip of the tongue perpendicular to the pterygomaxillary vertical line (TT-PMV) was not statistically significant.

**Conclusions:**

These preliminary cephalometric results indicate that BioEx can be an effective OFMFT modality in increasing the tonicity of the tongue muscles to establish more normalized tongue position at rest.

## RESUMO

**Objetivo:**

Introduzir protocolos de terapia miofuncional orofacial (TMO) recém-estruturados e desenvolvidos, denominados Bio-Exercise (BioEx), e avaliar o efeito do tratamento com esse método, usando análise cefalométrica em radiografias laterais de jovens com má oclusão e postura baixa da língua.

**Métodos:**

Foi realizado um estudo preliminar retrospectivo com registros ortodônticos de 28 pacientes (idade média de 8,41±1,45 anos, 13 homens, 15 mulheres) tratados com terapia BioEx usando elevadores de língua por 18,14±9,04 meses (intervalo: 6 a 37 meses). As radiografias cefalométricas laterais pré-tratamento (T_0_) e pós-tratamento com BioEx (T_1_) foram analisadas quanto às alterações na postura da língua, por meio de medidas lineares, anteroposteriores e verticais. Os dados foram analisados pelo teste *t* pareado, considerando-se um nível de significância de 5%.

**Resultados:**

O comprimento da língua (CL) e a altura da língua (AL) aumentaram entre T_0_ e T_1_, com significância estatística. A diminuição do dorso da língua perpendicular ao plano palatino (valor Td-PP) foi estatisticamente significativa. O aumento da ponta da língua perpendicular à linha vertical pterigomaxilar (PL-PMV) não foi estatisticamente significativo.

**Conclusões:**

Esses resultados cefalométricos preliminares indicam que o BioEx pode ser uma modalidade de TMO eficaz no aumento da tonicidade dos músculos da língua, para estabelecer uma postura de língua adequada em repouso.

## INTRODUCTION

The Academy of Orofacial Myofunctional Therapy and the Academy of Applied Myofunctional Sciences describe the disturbed and unbalanced orofacial muscular behaviors as orofacial myofunctional disorders.^1^ Orofacial myofunctional therapy (OFMFT) is, therefore, a form of physical therapy that enhances functionality of the orofacial muscles encompassing the dental and periodontal structures.^2^ ^,^ ^3^ Re-training or re-directing the muscular activities around the oronasal cavity can promote an environment that supports more stable and balanced occlusions as well as providing solutions to problems that result from weak and uneven biting forces.^1-3^

The orofacial musculature provides general boundaries to the dentition. The lips and the cheeks support the dentition from outside; and the tongue, from inside. The malfunction of the perioral musculature can create dental and periodontal problems. For example, when the lips are apart and the tongue is overly relaxed in rest, the imbalance between these muscles can affect the positioning of the teeth, resulting in malocclusions, such as the anterior open bite, or interdental spacings.^4^ In addition, the orofacial muscular imbalance can be associated with poor speech articulation problems or abnormal swallowing patterns.^1^ The purpose of OFMFT, therefore, is to optimize the pressure and the balance of the orofacial muscles that are in proximity of the dentition and the oronasal cavities, to alleviate such issues, based in the Functional Matrix Theory.^5^ Moss^5^initially proposed this theory in 1968, and continued to advocate the concept by revising it in 1990s. According to the theory, the growth and development of the orofacial structures are heavily influenced by various neurotrophic responses to the functional needs. The theory emphasizes that the soft tissues surrounding the facial skeletons are critical in signaling the responses to the growth and development of the hard tissues.^5^

Since the oronasal functions and the dentitions are closely related, OFMFT aims to guide and develop more harmonious occlusion by normalizing the four major oronasal functions - mastication, speech articulation, swallowing and breathing - through rigorous physical exercises of the tongue and the lip muscles.^1^ However, the clinical protocol of OFMFT has not been refined in the clinical practice, and current available protocols tend to demand very high level of cooperation from the patient for a long period of time. In addition, the physical exercise regimen is very complicated for the patient to follow at home and the care provider cannot verify the level of patient’s cooperation because there is no tracking system incorporated in the conventional OFMFT. Patients are, therefore, easily discouraged by the complexity of the therapy and tend to doubt the therapeutic values relative to the financial investment. 

Bio-Exercise (BioEx) is a novel OFMFT protocol that is patient-centered and, therefore, easy-to-follow at home. This is an outcome of our efforts to structure and systematize conventional OFMFT protocols while maintaining the high level of therapeutic efficacy. BioEx utilizes special functional appliances named “BioEx Xenium (BEX)” that were developed by Dr Kyu-Rhim Chung, the founder of Biocreative Orthodontic Strategies in 1980s.^6^ ^,^ ^7^ Thus, the present study aimed to introduce Bio-Exercise as a newly structured OFMFT protocols, and to determine the cephalometric and dimensional effects of this method, using lateral cephalometric analysis on malocclusion with low tongue posture in young patients.

## MATERIAL AND METHODS

### A. ELEMENTS OF BIOEX THERAPY

#### 1. Assessment of orofacial myofunctional disorder (OMD) for BioEx therapy

Assessment of orofacial myofunctional disorder for BioEx therapy is devised based on the reports by Suzuki et al.^8^ and Osamu et al.^9^ The BioEx OMD assessment is composed of four main categories: (1) Habits, (2) Lip and facial muscles, (3) Tongue and Swallowing muscles and (4) Anatomy. Within these four categories, there are seven topics to assess:

1) Documenting parafunctional oral habits such as thumb/finger sucking, tongue sucking, nail biting, bruxism, tongue thrusting, or others.
2) Recording the chewing styles such as the rubbing tendency of the tongue against the palate, excessive saliva, excessive facial contraction, noisy chewing or occlusal smacking, sticking out of the tongue in receiving the foods.
3) Assessing lip conditions such as upper lip length, lower lip strain, lip posture, and mentalis muscle at rest and at swallowing.
4) Measuring muscle tone of the right and left masseter muscles. Electromyogram can be added to quantify the muscle tone at this time.
5) Assessing the size and the position of the tongue, and to check if the lingual frenum is limiting the tongue movement. The tongue position recording is the most critical component of the assessment of Bio-Exercise Therapy. The tongue base and tongue tip are traced on the lateral cephalometric head film to record the tongue position before and after Bio-Exercise.
6) Recording the oropharyngeal condition related to swallowing. The size of palatine tonsils and adenoid vary with the patient’s age and it is very important to monitor them for the growing patients. In addition, the height and width of the hard palate, the length of the soft palate, gag reflex, as well as whether the teeth are apart during the swallowing are recorded here.
7) Checking any other relevant oronasal conditions such as temporomandibular disorders, allergy or nasal congestions, sleep-related breathing disorders, speech articulation issues, minimum area of the airway as seen in the cone beam computed tomography, and body posture.

### 2. BioEx and BioEx Xenium (BEX)

**Figure 1:**
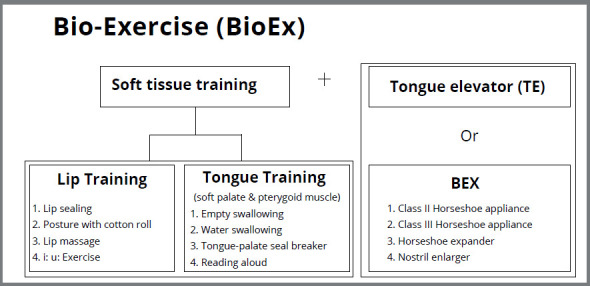
Schematic illustration of Bio-Exercise (BioEx).

The focus of Bio-Exercise is to stimulate the muscular activities around the oronasal cavities, which will improve and maintain the occlusion, as well as to provide a favorable environment for the normal development of the face (Fig 1). The establishment of tonic balance between the lips and the tongue provides an optimal pressure level for a healthy stable occlusion based on the individual needs of the patient. 

**Figure 2:**
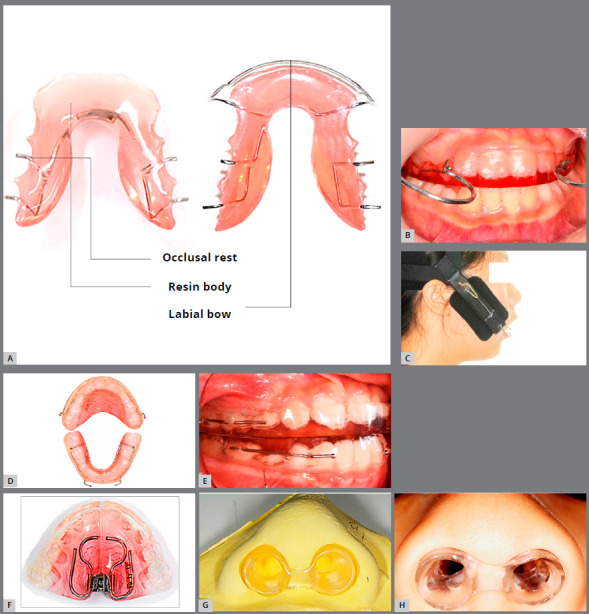
BioEx Xenium (BEX): **A**) Tongue elevator (TE) as main BEX; **B, C)** Class II Horseshoe appliance and high-pull J-hook headgear; **D, E**) Class III Horseshoe appliance and intraoral photo; **F**) Horseshoe expander; **G, H**) Nostril enlarger appliance

Therefore, BioEx primarily focuses on the trainings of the tongue and lips, and we call this as key soft tissue. One of the major differences that set Bio-Exercise apart from conventional OFMFT is that it utilizes unique removable orthodontic appliances that are designed to help stimulate the orofacial muscles (Fig 2). These appliances are called Bio-Exercise Xenium (BEX).^6^ ^,^ ^7^ ^,^ ^10^ ^-^ ^12^ BioEx is more effective when it is performed while keeping a BEX in the mouth. Examples of BEXs are tongue elevators (Fig 2A), Class II or Class III horseshoes (Figs 2B, 2C and 2D, 2E), horseshoe expanders (Fig 2F), or nostril enlargers (Figs 2G, 2H).^6^ ^,^ ^7^ ^,^ ^12^ ^-^ ^14^

A tongue elevator is one of the most frequently used BEXs and is composed of three primary components: resin body (Forestacryl, Forestadent co., Pforzheim, Germany), occlusal rest and auxiliaries (Fig 2).^6^ ^,^ ^11^ ^,^ ^12^ A tongue elevator lets the tongue rest on the resin body so that the tongue stays lifted at rest when it is placed in the mouth. The occlusal rests are incorporated in the resin body only when the mandibular molars will benefit from the intrusive force of the tongue’s weight. Auxiliaries include a labial bow, screen, and crib. These can be easily modified to custom fit a BEX to meet the patient’s specific needs. Therefore, a tongue elevator has three primary functions:1) lift the tongue effectively, 2) retain the teeth alignment post-orthodontic treatment, and 3) intrude the mandibular molars. 

#### 3. Lip training in BioEx (Lip BioEx)

**Figure 3:**
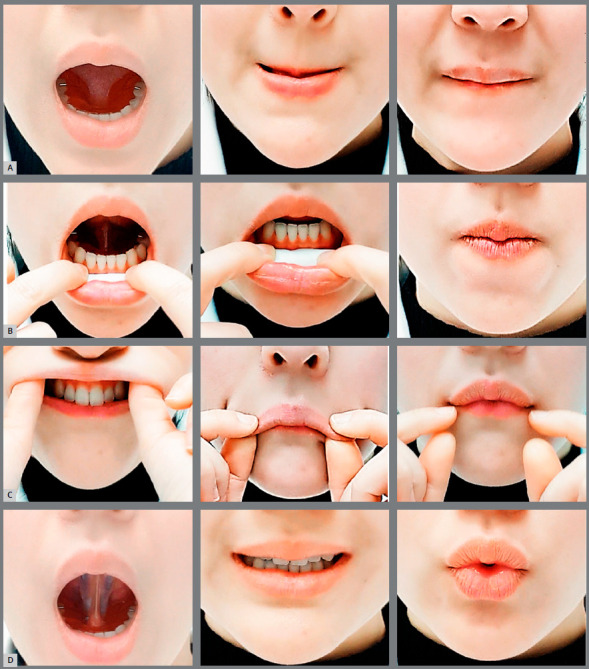
Lip BioEx Procedure. A) Lip sealing. B) Posture with cotton roll. C) Lip Massage. D) Exercises to pronounce the “I” and “U” sounds.

Lip BioEx is executed with a tongue elevator inside the mouth (Fig 3). 

*Lip sealing (Fig 3A)*

1) Place the tongue elevator inside the mouth. Make sure to let the tongue rest comfortably on the tongue elevator’s acrylic body.
2) Close the lips so that the lower lip covers the upper lip.
3) Slowly stretch the upper lip down using the lower lip.
4) Open the mouth to relax the lips and then repeat from the step (2)
5) Repeat 10 times a day.

*Posture with cotton roll (Fig 3B)*

1) Place the tongue elevator inside the mouth. Make sure to let the tongue rest comfortably on the tongue elevator’s acrylic body.
2) Place a cotton roll deep in the lower anterior labial vestibule.
3) Close the upper and lower lips together for 5 minutes while placing the tip of the tongue at the rugae of the anterior palate.
4) Perform this exercise once a day.

*Lip massage (Fig 3C)*

1) Place the tongue elevator inside the mouth. Make sure to let the tongue rest comfortably on the tongue elevator’s acrylic body.
2) Stretch down the upper lip to cover the lower lip using the thumb and index finger
3) Open the mouth to relax the lips and then repeat from the step (2)
4) Repeat 5 times a day

*Exercises to pronounce the “I” and “U” sounds (Fig 3D)*

1) Place the tongue elevator inside the mouth. Make sure to let the tongue rest comfortably on the tongue elevator’s acrylic body.
2) Lift the corner of mouth on both sides to make a long “I” sound.
3) Protrude the upper and lower lips as far out as possible to make a long “U” sound.
4) Repeat this exercise 10 times a day

**Figure 4:**
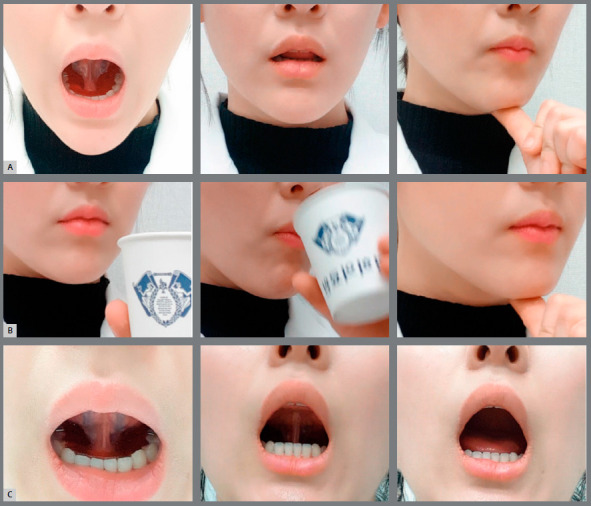
Tongue BioEx Procedure. A) Empty swallowing, B) Water swallowing, C) Tongue-palate seal breaker.

*4. Tongue training in BioEx (Tongue BioEx, Fig 4)*

The following is the BioEx protocol for the tongue training. Similar to the lip training exercise, BioEx for the tongue is executed with a tongue elevator inside the mouth.

*Empty swallowing (Fig 4A)*

1) Place the tongue elevator inside the mouth. Make sure to let the tongue rest comfortably on the tongue elevator’s acrylic body.
2) Push the chin up with a thumb.
3) Swallow with the tongue sealed on the palate as much as possible. It is optional to drink water to stimulate the swallowing motion at this step.
4) Repeat these steps in front of a mirror, 10 times a day.

*Water swallowing (Fig 4B)*

1) Place the tongue elevator inside the mouth. Make sure to let the tongue rest comfortably on the tongue elevator’s acrylic body.
2) Pour a small mouthful of water in the mouth and close the mouth to let the molars to contact without any lip restraints.
3) Push the chin up with a thumb.
4) Swallow with the tongue sealed on the palate as much as possible.
5) Make sure the lips do not contract during the swallowing.
6) Repeat these steps in front of a mirror, 10 times a day.

*Tongue-palate seal breaker (Fig 4C)*

1) Place the tongue elevator inside the mouth. Make sure to let the tongue rest on the tongue elevator’s acrylic body comfortably.
2) Seal the tongue on the palate and then make a short suction sound as quickly breaking the tongue seal from the palate.
3) Close the lips.
4) Repeat these steps 30 times a day.

*Reading aloud*

Place the tongue elevator inside the mouth. Make sure to let the tongue rest comfortably on the tongue elevator’s acrylic body.

Read a book out loud for 10 minutes once a week, while keeping a bite stick on the molars on each side simultaneously.

**Figure 5:**
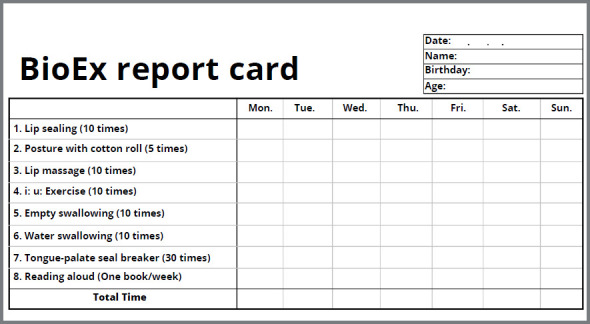
BioEx report card.

*5. BioEx report card (Fig 5)*

The full schedule of BioEx includes 10 times of lip sealing, 5 minutes of posture with cotton roll, 30 times of lip massage, 10 times of “I” and “U” sounds exercise, 10 times of empty swallowing, 10 times of water swallowing, and 30 times of breaking of the tongue seal. Duration usually takes approximately 20 minutes. Reading aloud is recommended once a week. The patient is given with a BioEx report card and is required to log in progress on a daily basis. The treating orthodontist should assess the report card on a monthly basis. A customized prescription of the BioEx components is recommended based on the BioEx-OMD assessment. For example, more repetition of tongue-palate seal breaker can be prescribed if a patient presents a low tongue resting position with a narrow maxillary arch.^17^ More repeated empty swallowing or water swallowing exercise is recommended to strengthen the lateral pharyngeal wall of patients with obstructive sleep apnea or chronic snoring.^18^ ^,^ ^19^

### B. PRELIMINARY CEPHALOMETRIC STUDY

#### 1. Subjects

A retrospective preliminary study with orthodontic records from 28 patients (mean age of 8.41±1.45-year-old, 13 males, 15 females) treated with BioEx therapy using tongue elevators was performed. Eligible individuals were all patients treated by BioEx in the orthodontic service. The total treatment time of BioEx was 18.14±9.04 months (range: 6 to 37 months). The Institutional Review Board of Kyung Hee University Dental Hospital approved the study (no. KH-DT19020). 

A power analysis using G*Power software (version 3.1.9.7; Franz Faul University, Kiel, Germany) determined that a sample size of 27 subjects per group would provide a power of 81% to detect significant differences with an 0.5 effect size and a value of 0.05. The inclusion criteria were growing patients with low tongue posture, who were cooperative on BioEx treatment with tongue elevator ( 20 min of BioEx treatment/day), combined or not with Horseshoe appliance, and available pre-treatment (T_0_) and post-BioEx treatment (T_1_) lateral cephalograms. 28 patients met the criteria: anterior open bite with skeletal Class I (n = 5), skeletal Class II (n = 13), and skeletal Class III malocclusion (n = 10). Patients with skeletal asymmetry, severe canting problem, a history of jaw surgery, tongue surgery, treated with speech therapy and/or medication, were excluded. 

#### 2. Cephalometric analyses of lateral head films

**Figure 6:**
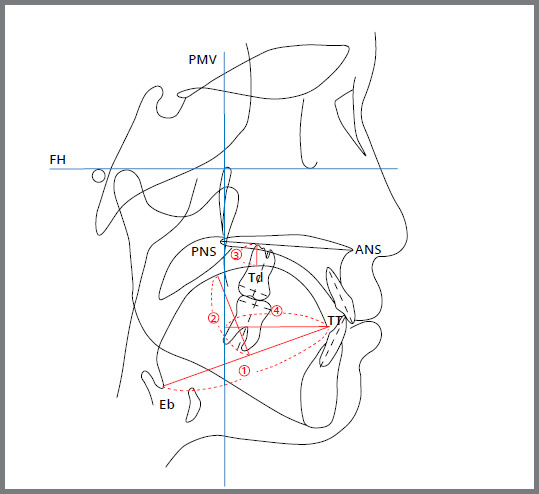
Schematic illustration of cephalometric measurements, 1) TGL (mm): tongue length (distance between Eb and TT), 2) TGH (mm): tongue height (maximum height of line perpendicular to Eb-TT line at tongue dorsum), 3) Td-PP (mm): distance along perpendicular from Td to palatal plane, 4) TT-PMV (mm): distance along perpendicular from TT to PMV line.

Patients were asked to relax their face and mouth, swallow once and hold the tongue in the position, immediately before taking the head film. The cephalograms were taken in natural head position with the centric relation of the mandible (FH plane parallel to the floor). One orthodontist traced all the cephalograms. This preliminary cephalometric study mainly focused on tongue-related variables’ changes. Four variables were measured to quantify the tongue position before and after BioEx (Fig 6): tongue length (TGL), tongue height (TGH), dorsum of the tongue perpendicular to the palatal plane (Td-PP), and the tip of the tongue perpendicular to the pterygomaxillary vertical line (TT-PMV).^15^

These data were confirmed by repeating the same data collection process two-weeks later. To verify the reliability of the measurements, intra-Class correlation coefficient (ICC) was calculated. The measured cephalometric data have proven to be reliable because the ICC derived was greater than 0.94 at 95% confidence interval.

#### 3. Statistical analysis

All statistical analyses were performed using SPSS 22.0 (SPSS, Chicago, IL, USA) to correlate the four variables related to the tongue position on the lateral head films. Discrepancies of the values of TGL, TGH, Td-PP, and TT-PMV before (T_0_) and after (T_1_) BioEx followed a normal distribution with *p*>0.05 using a Shapiro-Wilk test on the 28 subjects of the study. The mean and standard deviation of the values at T_0_ and T_1_ were recorded. Independent *t*-test was performed to evaluate sex difference considering a 5% significant level. To check for any significant differences in the measured items as recorded in two different measurements, a paired *t*-test was performed considering a 5% significance level.

#### 4. Results

There was no statistically significant difference between male and female group (*p*>0.5). TGL was defined as the distance between the base of epiglottis (Eb) and TT. The average TGL was 68.19±6.11mm at T_0_ and 73.43±7.30mm at T_1_, with the *t*-value of 5.19 and *p*<0.001 (Table 1). TGL increased statistically significantly between T_0_ and T_1_. TGH was defined as the longest distance between TGL and Td. The average TGH was 25.83±3.45mm at T_0_ and 28.13±3.63mm at T_1_ with the *t*-value of 3.96 and *p*<0.001. TGH also increased statistically significantly between T_0_ and T_1_. Td-PP was defined as the shortest distance between PP and Td. The average Td-PP was 7.77±2.95mm at T_0_ and 5.80±3.62mm at T_1_ with the *t*-value of -4.18 and *p*<0.001. The decrease of Td-PP value was statistically significant. TT-PMV was defined as the distance between TT and PMV. The average TT-PMV was 41.49±5.76mm at T_0_ and 43.18±5.86mm at T_1_. The increase of TT-PMV, however, was not statistically significant. 

**Table 1:** Comparisons of the T0 to T1 changes (paired *t*-test).

Variables	Mean ± SD (T0)	Mean ± SD (T1)	Mean ± SD (T1-T0)	t	p
TGL (mm)	68.19 ± 6.11	73.43 ± 7.30	5.24 ± 5.43	5.19	***
TGH (mm)	25.83 ± 3.45	28.13 ± 3.63	2.29 ± 3.12	3.96	***
Td-PP (mm)	7.77 ± 2.95	5.80 ± 3.62	-1.96 ± 2.54	-4.18	***
TT-PMV (mm)	41.49 ± 5.76	43.18 ± 5.86	1.69 ± 5.36	1.70	NS

NS: not significant, *p<0.05; **p<0.01; ***p<0.001.

## CASE REPORT

### CASE 1

**Figure 7:**
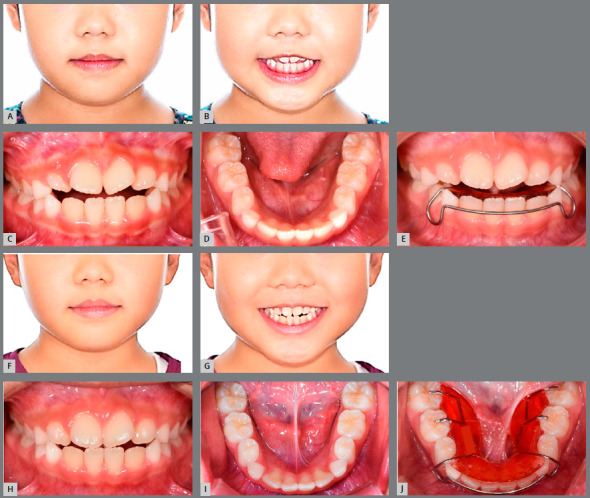
Case 1. **A, B, C,** D) Pretreatment extraoral and intraoral photographs. E) BioEx application with BEX ( Tongue Elevator ). **F, G, H,** I) Extraoral and intraoral photographs three months after BioEx. J) Lower occlusal view, with tongue elevator.

**Figure 8:**
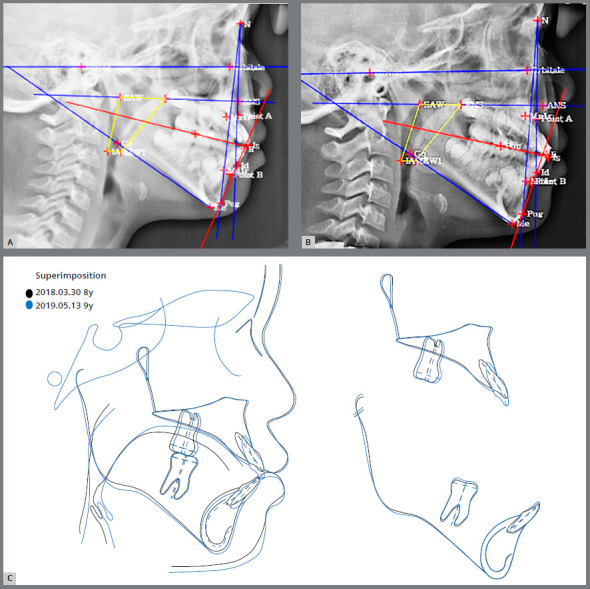
Case 1. Pretreatment (A) and 9 months after BioEx treatment (B) lateral cephalogram and Tweemac analysis. C) Superimposition of pretreatment and 9 months BioEx treatment cephalometric tracings.

An 8-year-and-6 months-old female child patient came in for orthodontic consultation with the chief complaint of *“My front teeth do not touch each other when I close my mouth”*. Clinical, radiographic, and photographic evaluation revealed that the patient presented Class I skeletal and dental patterns, localized dental rotation of the maxillary central incisors, anterior open bite, mouth breathing, low tongue posture, hypotonic upper lip, and short lingual frenum (Fig 7A-D, Table 2).^16^ A BioEx was prescribed using the tongue elevator (BEX) and lip training (emphasizing the 10 times of upper lip massage) and tongue training (emphasizing the 30 times of tongue-palate seal breaker) (Fig 7E). No BEX was used for the upper arch. The tongue elevator was recommended to be worn all the time. Even though the patient was a young child, she followed the BioEx regimen with excellent compliance because she was extremely motivated to correct the anterior open bite. The anterior overbite improved after 3 months of BioEx (Figs 7F-I and 7J). In addition, she was able to move her tongue to touch the anterior palate, which she was not able to do initially, due to the short lingual frenum (Fig 7J). The post-treatment facial photograph and lateral cephalogram demonstrate improvement of facial profile and inter-incisal relationship when compared with the pretreatment data (Figs 7F-I and 8). A decrease in lower face height contributed to the improvements. The lower esthetic angle was decreased from 21.6° to 18.2° (Figs 8A and 8B).^16^ The superimposition of initial and final cephalometric tracings confirmed improvement of facial development (Fig 8C).

**Table 2:** Cephalometric measurement of Case 1.

Variables			Pretreatment	Post-treatment	Mean	SD
Skeletal						
A-P position	N.perp-A (mm)		0.75	0.08	2.69	3.64
	N.perp-B (mm)		-2.93	-2.52	-1.75	3.9
Mx.-Mn relationship	PA-PB (mm)		3.67	2.61	4.44	2.49
Divergency	FH.PP (degrees)		1.93	1.58	2.15	2.32
	PP.MP (degrees)		32.92	33.64	30.28	4.27
	PP.OP (degrees)		12.12	11.63	11.17	2.48
	MP.OP (degrees)		20.81	22.01	19.72	3.53
Dental						
Axis of incisors	Upper	U1.PP (degrees)	130.38	119.56	115.31	4.85
	Lower	IMPA (degrees)	99.02	93.22	93.67	7.05
Dentoalveolar						
Convexity of lower symphysis	Esthetic angle (degrees)		21.57	18.19	14.31	5.65
Soft Tissue						
Airway	Upper airway width (mm)		23.59	19.63	25.57	2.74
	Lower airway width (mm)		6.87	6.68	11.57	3.92

N.perp-A = the line from the nasion perpendicular to the palatal plane to the point A. N.perp-B = the line from the nasion perpendicular to the palatal plane to the point B. PA-PB = distance by projecting points A and B perpendicularly to the palatal plane. FH-PP = palatal plane angle, angle between FH plane and palatal plane. PP-MP = angle between palatal plane and Me-Go line. PP-OP = angle between palatal plane and occlusal plane. MP-OP = angle between Me-Go line and occlusal plane. U1-PP = angle formed by the intersection of tooth axis of upper incisor and palatal plane. IMPA = angle between mandibular plane and mandibular incisor axis. Esthetic angle = chin angle, measured from nasion perpendicular to the palatal plane to the lower esthetic plane (infradentale-pogonion). Upper airway width = the distance between PNS and the posterior wall of the airway, parallel to PP. Lower airway width = the distance from the intersection of the anterior pharyngeal wall and the mandibular body to the posterior pharyngeal wall.

### CASE 2

**Figure 9:**
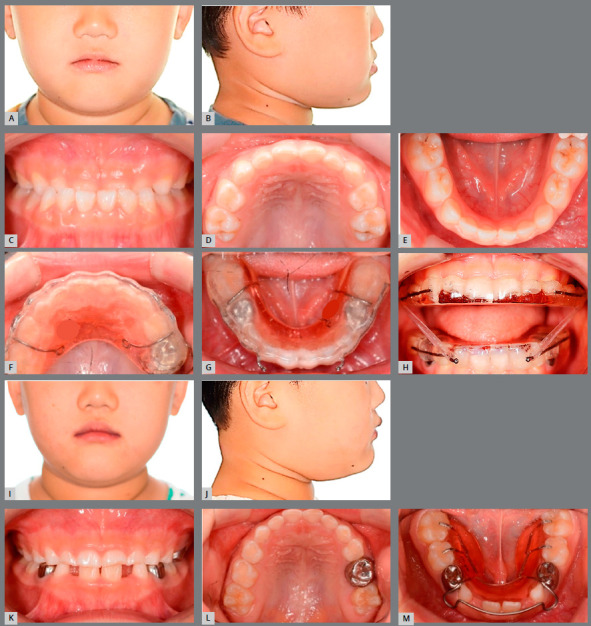
Case 2. **A-E**) Pretreatment extraoral and intraoral photographs. **F, G, H**) Class III BEX (Horseshoe appliance) application with BioEx. **I-L**) Extraoral and intraoral photographs 10 months after Class III Horseshoe appliance treatment. **M**) Lower occlusal view with the second BEX (tongue elevator).

**Figure 10:**
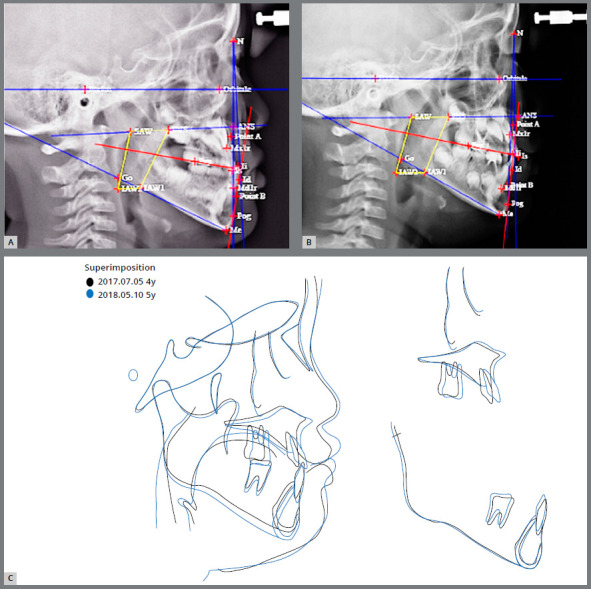
Case 2. Pretreatment (A) and 10 months after first BEX with BioEx treatment (B) lateral cephalogram and TWEeMAC analysis, C) Superimposition of pretreatment and 10 months BioEx treatment cephalometric tracings.

A 4-year-and-11-months-old male child patient was evaluated for an anterior crossbite in the full primary dentition (Fig 9A-E). The patient was diagnosed to have a skeletal Class III, dental Class III, shown as a mesial step occlusion of the primary dentition, anterior crossbite, large tongue with a thrusting habit, and snoring (Table 3). A BioEx was prescribed using a pair of Class III horseshoe appliances (Forestacryl, Forestadent Co., Pforzheim, Germany) as BEX to stimulate the maxillary protraction (Fig 9F-H). The patient wore the horseshoe appliance with 5/16” 2oz Class III interarch elastics full time. Ten-times of lip sealing was prescribed to strengthen the lower lip muscles. Ten-times of water swallowing exercise was also emphasized to train the tongue tip to touch the palate at rest and during swallowing. The anterior crossbite was corrected with 10 months of BioEx, including an elevated tongue position at rest (Fig 9I-L). In addition, the lower lip showed increased muscle tone. Following these corrections, the Class III horseshoe appliances and tongue elevator combination approach (20min BioEx/day) were replaced by a tongue elevator only, as a second BEX to continue to support the elevated tongue position long-term (Fig 9M). The 10 months BioEx treatment lateral cephalogram and superimposition demonstrate improvement of tongue posture and facial development, when compared with the pretreatment data (Fig 10). 

**Table 3:** Cephalometric measurements of Case 2.

Variables			Pretreatment	Post-treatment	Mean	SD
Skeletal						
A-P position	N.perp-A (mm)		-3.67	-0.99	1.94	3.87
	N.perp-B (mm)		-2.69	-4.82	-1.09	6.04
Mx.-Mn relationship	PA-PB (mm)		0.98	3.83	3.03	3.71
Divergency	FH-PP (degrees)		2.90	0.94	1.50	2.21
	PP-MP (degrees)		28.58	30.02	25.76	6.57
	PP-OP (degrees)		12.84	12.08	8.03	4.24
	MP-OP (degrees)		15.74	17.94	18.82	5.34
Dental						
Axis of incisors	Upper	U1-PP (degrees)	99.63	111.2	118	5.51
	Lower	IMPA (degrees)	75.2	78.28	91.91	5.43
Dentoalveolar						
Convexity of lower symphysis	Esthetic angle (degrees)		11.94	6.81	9.71	4.17
Soft Tissue						
Airway	Upper airway width (mm)		19.18	21.61	26.4	3.18
	Lower airway width (mm)		11.26	16.86	12.4	3.33

N.perp-A = the line from the nasion perpendicular to the palatal plane to the point A. N.perp-B = the line from the nasion perpendicular to the palatal plane to the point B. PA-PB = distance by projecting points A and B perpendicularly to the palatal plane. FH-PP = palatal plane angle, angle between FH plane and palatal plane. PP-MP = angle between palatal plane and Me-Go line. PP-OP = angle between palatal plane and occlusal plane. MP-OP = angle between Me-Go line and occlusal plane. U1-PP = angle formed by the intersection of tooth axis of upper incisor and palatal plane. IMPA = angle between mandibular plane and mandibular incisor axis. Esthetic angle = chin angle, measured from nasion perpendicular to the palatal plane to the lower esthetic plane (infradentale-pogonion). Upper airway width = the distance between PNS and the posterior wall of the airway, parallel to PP. Lower airway width = the distance from the intersection of the anterior pharyngeal wall and the mandibular body to the posterior pharyngeal wall.

## DISCUSSION

One of the most important components of OFMFT is how to re-train the tongue muscles to learn the most physiologically harmonious position at rest, and stabilize the learned tongue position or the reprogramed tongue muscle whenever it returns to rest.^1^ ^-^ ^4^ Patients with chronic blockage of the nasal airway by rhinitis, tonsils, or adenoid tend to keep the tongue away from the palate to be able to breathe through the mouth. In addition, hypotonicity of the tongue muscles is another common finding on these patients. Therefore, the tongue muscle training often becomes the primary focus of OFMFT strategies. BioEx is mainly focused to encompass the spectrum from the comprehensive assessment to the patient-oriented self-reporting mechanism, which helps BioEx become an integral part of the full scope of orthodontic and dentofacial orthopedic treatment planning. A tongue elevator is one of the most commonly used BEXs in BioEx because it is designed to significantly elevate the tongue to adjust the tension of the tongue muscles at rest.^6^ ^,^ ^7^ ^,^ ^10^ ^-^ ^12^ At the same time, it provides an environment that can stimulate more effective training of the orofacial muscles. When occlusal stops are included in the tongue elevator design, the tongue resting on the tongue elevator can intrude the mandibular molars effectively reducing the mandibular plane angle and anterior open bite. 

Teeth tend to erupt following the path of least resistance between the inside and outside of the mouth. The outside of the dentition is mostly controlled by muscular movements of the orbicularis-oris and buccinators. The inside of the dentition is influenced by muscular movements of the tongue. In a case where a patient has to breathe through the mouth due to severe nasal airway resistance, the orbicularis oris muscle becomes hypotonic, allowing the lips to stay apart most of the time. This lets the tongue muscle dominate the equilibrium that determines position of the teeth, causing an anterior open bite by flaring the mandibular teeth out. An effective OFMFT should become an essential part of the active orthodontic treatment plan for this patient because it can strengthen the orbicularis-oris muscle and other facial muscles, which can augment the orthodontic treatment effects and assure a long-term post-treatment occlusal stability. In 1990, Thüer and Ingervall^20^ reported treatment effects of an oral screen in training the lip muscles. Sixteen children of ages between 7 and 11 years-old participated in the lip muscle training program. The muscular tension was measured using a dynamometer. When the water-filled system inside the lips was checked against the extraoral tension during resting, mastication, and swallowing, the strength of orbicularis oris muscle increased. The overjet decreased and the maxillary arch length decreased. The pressure on the lower lip during mastication increased temporarily during the treatment. In 1984, Owman-Moll and Ingervall^21^ reported an active lip muscle training for patients with lip incompetency that can help lengthening the lips and improving the lip movement. In 1998, Taner-Sarivov^22^ reported that lip sealing exercise can enhance the functional environment for the mouth movements. A study published in 2009 reported that patients who go through adenotonsillectomy with chronic mouth breathing habit are recommended to include exercises of orbicularis oris muscle as crucial part of post-surgical care regimen.^23^

The tongue plays a critical role in various physiological functions such as respiration, mastication, swallowing and speech.^6^ During the normal swallowing, the tip of the tongue rests at the alveolar ridge of the anterior palate and the body of the tongue rises gradually toward the posterior palate. Many malfunctions of oral musculature are often closely related to the low resting tongue position, large tongue size, uncoordinated swallowing pattern, and oral habits such as anterior or lateral tongue thrusting and tongue scalloping.^24^ In 2015, Moschik et al.^25^ studied the effects of a series of swallowing exercise using a gelatin pad on the palate with the goal of improving the tongue position while swallowing. This study reported increased intercanine distance of the maxillary dental arch by 3.2mm, increased length of the philtrum by 5.4mm, and decreased overjet by 1.2mm. It is interesting to note that the increase of the intercanine distance resulting from the swallowing exercise was shown in both adult and growing patient groups. This might be due to the combined efforts of the tongue muscles, orbicularis oris muscles and buccinator. The contraction of the orbicularis oris tends to incline the maxillary anterior teeth palatally and the tongue influences to widen the intercanine distance of the maxillary arch. In 2015, Van Dyck et al^26^ measured the tongue lifting strength among two groups of children who received myofunctional therapy (MFT) and who did not, when they had the anterior open bite with abnormal tongue movements. They used an IOPI tongue pressure detector for the study and the MFT was composed of 20 sessions with increased level of intensity at each session. Clinical findings reported that the group who received MFT showed an increase of strength when lifting the tongue as the therapy progressed. When the tongue is placed anterosuperiorily in rest, the tongue pressure on the palate is constant, which in turn can effectively change the horizontal and vertical relationships of the occlusion. However, whether the anterior posture of the tongue is the cause to the anterior open bite or vice versa is still a topic of discussion and needs to be determined with further investigation.

The cephalometric analysis of 28 children who went through BioEx as part of their orthodontic growth modification treatment in this preliminary study showed statistically significant increases of TGL and TGH, as well as decreases of Td-PP. These results indicate that BioEx can be an effective OFMFT modality in increasing the tonicity of the tongue muscles to establish more normalized tongue position at rest. It is also important to note that the tongue was retained much closer to the palatal arch at rest following BioEx treatment. The weakness of OFMFT research still lies in the fundamental difficulties related to unstandardized study methodologies, multifactorial etiologic nature of large spectrum of muscular dysfunctions, lack of quantification tools for cooperation for OFMFT regimen at home, and the lack of randomized clinical trials. Efforts to overcome such difficulties are necessary to produce more effective and predictable treatment outcomes using various OFMFT. 

We compared the only tongue-related variables changes in the young patients with various kinds of skeletal problems in this study. Further studies about the effects of BioEx in various patient groups and long-term stability are needed to understand more about the differences between BioEx and conventional OFMFT approaches.

## CONCLUSIONS

Bio-Exercise (BioEx) is an innovative structured and simplified OFMFT that utilizes BEXs to re-establish the balance among orofacial muscles, with the ultimate goal of improving the skeletal and/or dental relationships. Five elements of BioEx encompasses the spectrum from the comprehensive assessment to the patient-oriented self-reporting mechanism, which helps BioEx become an integral part of the full scope of orthodontic and dentofacial orthopedic treatment planning. This preliminary cephalometric study showed that BioEx affect tongue posture and activity significantly.
